# TiO_2_-Based Nanostructures, Composites and Hybrid Photocatalysts

**DOI:** 10.3390/ma15041271

**Published:** 2022-02-09

**Authors:** Stefano Lettieri, Michele Pavone

**Affiliations:** 1 Institute of Applied Sciences and Intelligent Systems “E. Caianiello”, Consiglio Nazionale delle Ricerche (CNR-ISASI), Complesso Universitario di Monte S. Angelo, Via Cupa Cintia 21, 80126 Napoli, Italy; 2 Department of Chemical Sciences, University of Naples “Federico II”, Complesso Universitario di Monte Sant’Angelo, Via Cupa Cintia 21, 80126 Napoli, Italy; michele.pavone@unina.it

The field of materials sciences has always been strongly interconnected with the most significant technological developments in the modern era, and such an interconnection is absolutely evident at least since the 1950s revolution of electronics and microelectronics, driven by advances in the science of semiconductors.

Nowadays, there is a widespread awareness, not just in the scientific world but also in the applicative-industrial world, that the study of some physical and chemical–physical processes of a fundamental nature is not a matter relegated to specific sectors of academic research. In this statement, we refer in particular to some of the topics that are the subject of the Special Issue "*TiO_2_-Based Nanostructures, Composites and Hybrid Photocatalysts*”, edited by us [1]. These topics broadly involve the science and technology of titanium dioxide (TiO_2_) and its modifications (e.g., doping and TiO_2_-based composites) and lie at the core of research on photocatalytic materials. A topical list is shown in the web page of the Special Issue. Here, for the sake of simplicity, we list some of these topics along with few representative references:

- Fundamental properties of TiO_2_ nanostructures such as: electronic states, defects, optical properties, etc. [2,3,4,5,6].
- Applications of TiO_2_-based photocatalytic systems: water and/or air remediation [7,8,9], gas sensors [10,11,12,13], generation of solar fuels and energy applications [14,15,16,17], degradation of dyes and/or pharmaceuticals [18,19,20].
- Improvement of TiO_2_ photocatalytic efficiency through doping and/or self-doping [21,22,23,24] and/or through synthesis and use of TiO_2_-based composites/heterostructures [25,26,27].

The above topics address the fundamental questions that define the core of the science and technology not only of TiO_2_ but, more generally, of photocatalytic materials. These materials are a representative example of the interconnection between some branches of materials sciences and important technological challenges. In fact, the photo-transformative properties of nanostructured photocatalysts are relevant for two of the most important challenges of the modern era: water pollution and the depletion of fossil fuels.

Regarding water pollution, the availability of drinking water free of pathogens and of polluting/toxic chemicals has become a severe problem in many parts of the world and in developing countries. Hazardous contaminants can be removed from fresh water or be transformed in harmless species via oxidation processes: photodegradative oxidation promoted by TiO_2_ (or TiO_2_-based composites) is widely regarded as a possible route to pursue that goal. In fact, TiO_2_ in aqueous solution acts as an oxidative agent due to its ability to generate reactive oxygen species (ROS) under ultraviolet (UV) illumination. ROS species, in turn, function as bactericide agent and/or depolluting agent in fresh waters and wastewaters.

The depletion of fossil fuels and the environmental risk related to greenhouse gas release—also referred to as climate change—are also sources of social concerns. Public policies are, in fact, targeting the “carbon neutrality” goal in several ways, intended as the balance between emitting carbon into the atmosphere and absorbing carbon from the atmosphere to appropriate sinks (i.e., carbon sequestration). To achieve the goal, sustainable forms of energy exploitation need to be developed, hence the keyword of “energy transition” [28]. Additionally, in this regard, TiO_2_-based (photo-)catalysts play a crucial role in hydrogen and fuel production and in the carbon-free conversion of chemical energy into electrical energy.

Apart from its photocatalytic properties, TiO_2_ also exhibits chemo-resistive and chemo-optical effects, i.e., molecules adsorbed on its surface induce a redistribution of mobile charge and a surface band-bending, thus affecting the electrical conductivity [29] and the photoluminescence [30] of the material. This effect allows its use as the sensitive element of chemical (gas) sensors, as in some of the papers published in the Special Issue. 

Both photocatalytic and gas-sensing effects are relevant for practical purposes only when using materials with large values of the specific surface area (SSA). For this reason, TiO_2_ is consistently used in the form of either nanoparticle films/powders or of isolated nanostructures, deposited/synthesized via various chemical and physical methods that allows us to achieve SSA values of ~100 m^2^/cm^3^ [31,32,33,34,35]. While nanoparticle films/powders are easier to produce, quasi one-dimensional structures such as nanowires/nanopillars with diameters of a few tenths of nanometers can, in principle, be more effective for high-sensitivity gas sensors. This is because typical depletion width caused by adsorption is also of the order of a few tenths of nanometers, so that the presence of the analyte can in principle completely “pinch off” the charge carriers. In this context, the literature on the use of 1D and quasi-1D TiO_2_ structures for gas sensing has been reviewed by Kaur and coauthors in their review contribution [36] to the Special Issue.

Under these premises, covering the state-of-the-art of applications of TiO_2_ clearly represents a very demanding task, which has been excellently fulfilled by several published reviews in recent literature [37,38,39,40,41]. The review published by Lettieri, Pavone, and coworkers in this Special Issue [2], instead, focuses on specific *fundamental mechanisms* and processes that lie behind and determine the photo-physical behavior of the material. In particular, we refer here to issues such as the energy levels of occupied and excited defect (trap) states, optical absorption and photoluminescence, trapping and detrapping processes and lifetimes, while also dealing with attention with the interaction between photo-generated carriers and environmental oxygen (O_2_). Although often given for granted and not fully understood, these features are very important as they ultimately affect the functional performances of TiO_2_-based materials.

The pros and cons of TiO_2_ as photocatalytic agent are well established. Its two most important limitations are the impossibility to activate it by means of solar light and the overall low photoactivity. The latter is due to the fact that most of the photogenerated free carriers recombine before reaching the surface and reacting with the species to be transformed, e.g., the adsorbed H_2_O molecules in oxygen evolution reaction (OER). 

Possible approaches for overcoming these limitations can be classified in two categories: (1) routes based on doping of TiO_2_ and (2) routes based on heterojunction photocatalysts, where TiO_2_ is electronically coupled with a second material that acts as sensitizer or as cocatalyst.

Some of the papers published in the Special Issue deal with doped TiO_2_ [42,43,44]_._ Dopants in TiO_2_ can shrink the optical bandgap by introducing additional energy states in the semiconductor bandgap [45,46], thus allowing the generation of mobile charge carriers via absorption of sunlight. Moreover, dopants can favor the charge separation due to formation of Schottky junctions or can act as co-catalysts [26]. These modifications can all, in principle, enhance the photocatalytic efficiency.

Edelmannova and coauthors investigated the effect of lanthanide dopants (lanthanum and neodymium) on the photocatalytic generation of H_2_ from ammonia (NH_3_), reporting on a beneficial effect on the photocatalytic NH_3_ to H_2_ conversion associated by small concentrations (0.1 % weight) of Lanthanum (La) as dopant of TiO_2_ immobilized on foams [42]. 

Sturini and coauthors [43] reported on the use of composites made from pristine and nitrogen-doped TiO_2_ (N-TiO_2_) and sepiolite and zeolites for the photocatalytic removal of ofloxacin (a widespread antibiotic), remarking that, in this case, the presence of the dopant is not necessarily is benefit as the doping routes improve the optical absorption (as discussed above) but also decrease the overall specific surface of the immobilized photocatalyst. 

Finally, Gao et al. performed first-principle calculation of density of states modification caused by the presence of atoms belonging to the platinum family (Pd, Ru, Rh) as dopants of anatase TiO_2_ (101) surfaces, calculating the possible formation of occupied states above valence band edge and below the center the band-gap region, associated with Ru and Rh atoms [44]. These results can provide a support for the interesting experimental results on the photocatalytic performances of Ru-doped TiO_2_ [47].

As mentioned above, TiO_2_ also has an application in the gas sensors field. The use of 1D and quasi-1D TiO_2_ structures as gas sensors is reviewed by Kaur and coauthors [36]. We recall their review for an extensive bibliography on experimental results recorded for gas sensing devices toward different analytes, including both reducing species (e.g., H_2_, H_2_S, NH_3_, CO, ethanol, acetone) and oxidating species (e.g., NO_2_, O_2_).

TiO_2_ /O_2_ interaction and its exploitation for optical sensing is discussed in some detail in the review paper [2], also showing that mixed-phase TiO_2_ can be employed in an unconventional ratiometric approach based on the difference between photoluminescence of rutile and anatase phases [48].

The paper by Fioravanti and coauthors also deals with the use of TiO_2_ as chemical sensors. In particular, their work discusses the interesting application of TiO_2_/SnO_2_ composites in monitoring the degradation of hydraulic oils [49]. The authors used solid solutions of TiO_2_ mixed with SnO_2_ to fabricate thick-film resistive devices sensitive to the presence of the hydraulic fluids into the oil headspace, finding that optimal results, in terms of best responses and lower recovery time, were obtained with composites made by 90% (molar percentage) TiO_2_. 

Other works deal with preparation of various forms of TiO_2_ and application as photocatalysts, as briefly summarized below.

Di and coauthors [50] report on a hydrothermal procedure for the synthesis of self-assembled of anatase TiO_2_ microspheres of average diameters in the range of 0.5–3 um. Interestingly, they showed that the investigated chemical process employed allowed to tune the percentage of crystalline (001) facets, which is an important parameter, as highlighted by different studies showing that the exposure of well-defined crystalline anatase facets is accompanied by a significant boost in photocatalytic activity [25].

The work by Cizmic and coauthors [51] deals with the relevant problem of water pollution by pharmaceuticals. As the authors point out, conventional wastewater treatment plants are not designed for removing complex organic compounds, such as pharmaceuticals. In particular, the dispersion of antibiotics is a major issue, as it aggravates the spread of new branches of microorganisms resistant to conventional to antibiotics (antibiotic resistance). Cizmic and coworkers studied the ability of anatase TiO_2_ nanopowders to degrade (photo-oxidize) the azithromycin antibiotic, studied the influence of several parameters (e.g., water pH, spectral range of the UV light that activates the TiO_2_ photocatalysts, presence of sulfamethoxazole as interfering species) on the photodegradation, finding encouraging results and optimal condition at pH 10 under UV-C illumination.

M.G. Toro and coauthors [52] face the interesting challenge of incorporation of TiO_2_ nanopowders on woven fabrics. TiO_2_ incorporation can provide antibacterial and self-cleaning functions to fabrics, but the anchoring of particles on any flexible substrate is, generally speaking, a challenging issue. In the particular case of fabrics, irregular shapes of fibers often hamper the interfacial adhesion. The authors report the successful preparation of photocatalytic paper by preparing TiO_2_ hydrosols and introducing them in the production process of paper sheets. The use of sodium alginate as a binding agent proved to be beneficial for the mechanical properties (i.e., better tensile index, breaking length, tear and burst index, and air permittivity) of the photocatalytic paper, thanks to the interaction between hydroxyl groups on TiO_2_ surface, sodium alginate and cellulose. Finally, they also proved that photocatalytic papers can be used repeatedly while retaining their photocatalytic activity.

As a final consideration, we wish to point out that, as the literature on TiO_2_-based systems is so comprehensive, editorial products such as reviews and Special Issues are extremely valuable and useful for scholars (in particular PhD students who are approaching the field), provided that they focus on specific and, eventually, fundamental topics. We also believe that the knowledge that has being accumulating on TiO_2_-based systems will turn out to be very important even if other materials and systems will turn out to be more useful or even keystones for future breakthroughs. 

## Data Availability

Not applicable.
